# Combined Use of External Iliac Lymph Node Count and Bone Scintigraphy for PJI Diagnosis: A Prospective Study

**DOI:** 10.3390/diagnostics14222502

**Published:** 2024-11-08

**Authors:** Haotian Zhou, Yaji Yang, Jia Li, Qianshui Hu, Feilong Li, Leilei Qin, Wei Huang, Hai Wang, Qiang Cheng

**Affiliations:** 1 Department of Orthopaedic Surgery, The First Affiliated Hospital of Chongqing Medical University, Chongqing 400016, China; 18723512282@163.com (H.Z.); yaji-yang@stu.cqmu.edu.cn (Y.Y.); qianshui_hu@163.com (Q.H.); 152053@hospital.cqmu.edu.cn (F.L.); qinleilei@stu.cqmu.edu.cn (L.Q.); huangwei68@263.net (W.H.); 2 Chongqing Municipal Health Commission Key Laboratory of Musculoskeletal Regeneration and Translational Medicine, Chongqing 400016, China; 3 Orthopaedic Research Laboratory, Chongqing Medical University, Chongqing 400016, China; 4 Department of Radiology, The First Affiliated Hospital of Chongqing Medical University, Chongqing 400016, China; tianwei19900911@163.com; 5 Department of Orthopedics, Chongqing University Fuling Hospital, Fuling District, Chongqing 408000, China

**Keywords:** periprosthetic joint infection, external iliac lymph node, bone scintigraphy, diagnosis, inflammatory, immunology

## Abstract

**Background:** The reactive enlargement of external iliac lymph nodes and increased blood flow in the infected region are commonly observed in lower limb infections. We aimed to differentiate between aseptic loosening and periprosthetic joint infection (PJI) after joint replacement surgery by quantifying the number of enlarged external iliac lymph nodes and using bone scintigraphy to monitor blood flow. **Methods:** We recruited 124 patients undergoing revision surgery for aseptic loosening or PJI. All the patients underwent preoperative dual-energy computed tomography (DECT) imaging for external iliac lymph nodes and bone scintigraphy. The diagnostic value was evaluated using ROC curve analysis. **Results:** The number of enlarged external iliac lymph nodes was significantly higher in the PJI group than in the aseptic failure group (4.0 versus. 1.0, *p* value < 0.001). The median affected/unaffected side ratio in the blood pool phase of ECT in the PJI group was 1.49, significantly higher than the aseptic failure group’s median ratio of 1.04 (*p* value < 0.001). The AUC for diagnosing PJI using the number of enlarged lymph nodes alone was 0.91, and when using the bone scintigraphy blood pool phase alone, the AUC was 0.89. When both metrics were combined, the AUC increased to 0.95, which was higher than the AUCs for the ESR (AUC = 0.83), CRP (AUC = 0.76), and synovial fluid PMN% (AUC = 0.62). **Conclusions:** Combining the enlargement of the lymph node count with the bone scintigraphy blood pool phase is a promising approach for diagnosing PJI.

## 1. Introduction

Periprosthetic joint infection (PJI) following joint replacement surgery is a significant challenge in orthopedics [1]. The timely and accurate diagnosis of PJI is crucial for understanding its progression and prognosis. However, the biological characteristics of pathogens and individualized inflammatory responses in patients present substantial challenges in diagnosing PJI [2]. Traditional diagnostic tools, such as molecular markers like CRP and ESR, are easily accessible and inexpensive but lack specificity and have limited diagnostic accuracy [3,4]. Although tissue culture provides the strongest evidence for a PJI diagnosis, it frequently experiences a low positive rate and a high rate of false negatives [5]. Innovative molecular techniques, such as Polymerase Chain Reaction and Metagenomic Next-Generation Sequencing, have improved diagnostic accuracy, but they are often expensive and difficult to implement on a large scale [6,7]. As a result, we urgently need a readily available, rapid, and highly effective diagnostic method.

The lymphatic system is a crucial part of the body’s immune defense, playing a key role in combating and eliminating invading pathogens [8]. Lymph nodes are highly structured lymphoid organs located at lymphatic pathway junctions [9]. When pathogens invade the body, resident macrophages within the lymph nodes activate, swiftly coordinating local immune responses to eliminate microbes and their products from the lymph, strengthening the barrier functions of both the lymph and the nodes. Meanwhile, lymph fluid from the extracellular space carries antigen-presenting dendritic cells and macrophages into the lymph nodes through afferent lymphatic vessels, triggering humoral and cellular immune responses to clear the infection [10,11]. Ultimately, this immune response enlarges several lymph nodes, with changes in size and number closely correlated with the progression of the infection [12]. When local tissues are infected, the adjacent lymph nodes enlarge within 2–3 days, typically returning to their normal size within 3–4 weeks after the infection has been controlled [13]. In our earlier research, ultrasound monitoring of inguinal lymph node size revealed much more pronounced node enlargement in PJI patients compared to those with aseptic failure [14]. However, as the inguinal lymph nodes do not directly drain the lymph from deep pelvic and hip tissues, their effectiveness in diagnosing PJI after hip replacement surgery may be limited. Thus, we aim to identify drainage regions more directly linked to the hip joint and its surrounding tissues. By monitoring the number of enlarged lymph nodes in these groups, we hope to establish a more objective and accurate approach for detecting PJI following hip replacement surgery.

External iliac lymph nodes act as a “sentinel” for the monitoring of lower limb infections. Positioned along the external iliac artery, they are one of the lymph nodes in the pelvic region [15]. They receive lymphatic drainage from the femoral lymph nodes, inguinal lymph nodes, and the pelvic region [15]. External iliac lymph nodes not only reflect changes in the inguinal lymphatic drainage area but also monitor infections in the hip joint and deep pelvic tissues [16]. Soriano et al. suggested that the enlargement of the iliac lymph nodes in patients following hip replacement surgery is a strong predictor of PJI. [17] However, this study defined lymph node enlargement based only on the relative sizes of bilateral nodes, lacking a clear absolute size standard, risking misinterpretation. In other areas, such as oncology, studies have demonstrated that a lymph node short-axis diameter greater than 5 mm often indicates potential pathological changes [18]. Additionally, diagnosing infectious diseases based on a single enlarged lymph node may introduce selection bias, as nodes within the same group may display heterogeneity due to differences in cell populations and soluble factors [19]. Moreover, the lymph node response to infection is usually based on a collective effect, rather than just the antibacterial function of a single node. Therefore, this study quantifies the number of enlarged external iliac lymph nodes (short-axis > 5 mm) in PJI patients and those with aseptic loosening, and it also analyzes whether the number of enlarged nodes could serve as a novel diagnostic tool for PJI. For the precise quantification of the size and number of external iliac lymph nodes, we employed artifact-reducing dual-energy computed tomography (DECT) scanning, which can significantly mitigate metal artifacts that interfere with lymph node counting and measurement.

We note that using external iliac lymph node enlargement as an indicator of PJI following hip replacement surgery requires us to rule out other causes of enlargement, such as non-joint-related tissue inflammation or individual variations like mild pelvic inflammation (asymptomatic) or congenital lymphatic abnormalities, as the pathological enlargement of lymph nodes is closely related to blood and tissue fluid exchange in the drainage region [13]. Therefore, we will incorporate a diagnostic method that directly reflects the local blood and tissue fluid dynamics around the prosthetic joint. This technique utilizes bone scintigraphy with 99mTc-labeled phosphate compounds. Once injected into the body, the radiolabeled compound 99mTc is deposited in bone via ion exchange, chemical adsorption, and binding to the organic matrix of the bone [20]. By monitoring the radiation emitted by the radiopharmaceutical, this method tracks both systemic bone metabolism and local blood flow and inflammation around the prosthetic joint [20]. Compared to conventional imaging techniques like X-ray, CT, and MRI, bone scintigraphy demonstrates high diagnostic sensitivity for PJI, particularly in its early stages [21]. However, current research typically evaluates bone scintigraphy results through visual analysis, identifying an infection by visually observing increased radiotracer uptake on the generated images. Consequently, subjective factors may affect the final diagnostic outcome [22]. Furthermore, quantitative parameters and diagnostic thresholds for bone scintigraphy are currently lacking.

Currently, several authoritative organizations, such as the Musculoskeletal Infection Society (MSIS) and the American Academy of Orthopaedic Surgeons (AAOS), have proposed numerous diagnostic criteria for PJI, though none of them include imaging examinations as a primary diagnostic criterion. Bone scintigraphy, which localizes areas of inflammation, combined with counting the number of enlarged external iliac lymph nodes, may provide a promising diagnostic method for PJI. Therefore, by conducting this prospective controlled study, without incurring additional costs or harm to patients, we aimed to undertake the following: (1) validate whether the quantitative diagnostic thresholds of bone scintigraphy and the count of enlarged external iliac lymph nodes could accurately differentiate PJI; (2) optimize the combined application of bone scintigraphy’s quantitative diagnostic thresholds and external iliac lymph node counts for diagnosing PJI, aiming to further enhance diagnostic accuracy.

## 2. Materials and Methods

### 2.1. Patient Cohort and Characteristics

Patients were categorized into either the aseptic loosening group or the infection group following the 2013 Musculoskeletal Infection Society (MSIS) criteria, which we used to diagnose PJI [23]. This prospective cohort study, conducted from January 2021 to June 2024, included 124 patients who underwent total hip revision surgery due to PJI or aseptic failure. All patients signed informed consent forms before participating, and this study was approved by our hospital’s ethics committee (No. 2021-258) and registered with the Chinese Clinical Trial Registry (No. ChiCTR2100050785).

Patients were excluded from this study if they had any of the following: any type of skin ulcer, hematoma, or other skin infection within the past 2 weeks; recent lower limb trauma, surgery, or dislocation within 2 weeks; inflammatory arthritis (such as rheumatoid arthritis or gout); extra-articular lower limb infections or sexually transmitted diseases; malignancies that could cause lower limb lymph node changes (such as a history of lymphoma, gynecological cancers, lower limb skin cancer, or bladder cancer); vascular diseases of the lower limbs (e.g., deep vein thrombosis, embolism, or arterial and venous inflammation); individuals receiving immunosuppressive therapy; allergy to radiographic contrast agents; severe renal insufficiency; pregnant or breastfeeding women; or individuals who had undergone other nuclear medicine examinations in the past month. Fourteen patients were excluded: 3 cases of rheumatoid arthritis, 2 cases of gout, 2 cases of athlete’s foot, 1 case of prostatitis, 1 case of lower limb skin ulcer, 3 cases of malignancy (1 lymphoma, 1 cervical cancer, 1 prostate cancer), 1 case of allergy to radiographic contrast agents, and 1 patient who had undergone nuclear medicine imaging within the past month.

Baseline data, including age, gender, and BMI, were recorded for all patients. Venous blood was collected after admission to measure the serum erythrocyte sedimentation rate (ESR) and C-reactive protein (CRP). Before revision surgery, synovial fluid was collected for polymorphonuclear neutrophil percentage (PMN%) analysis. During revision surgery, at least three tissue samples were taken from the joint area for microbial culture (24–48 h and extended to 14 days).

### 2.2. Image Acquisition and Analysis

#### 2.2.1. Dual-Energy CT (DECT)

All patients were scanned in the supine position, with arms raised, thighs internally rotated, and toes touching. A Somatom Definition Flash (Siemens Healthineers, Erlangen, Germany) was used for DECT scanning of the pelvis and inguinal region. Scanning parameters were set as follows: a pitch of 0.6, a rotation time of 0.4 s, a slice thickness of 0.6 mm, and tube voltages of 80 kV and 140 kV. Three radiologists with orthopedic imaging experience independently reviewed the images of the external iliac lymph nodes on the affected side without the knowledge of the patients’ clinical details. They recorded the short-axis diameter and counted the number of lymph nodes with short-axis diameters ≥ 5 mm. Any differences in evaluation were resolved through discussion.

#### 2.2.2. Bone Scintigraphy Image Acquisition

The acquisition and analysis of bone scintigraphy images were performed in accordance with the current guidelines of the European Association of Nuclear Medicine (EANM) [24]. For 99mTc-MDP bone scintigraphy, a dual-head gamma camera (Discovery NM670, GE Healthcare, Chicago, IL, USA) with a low-energy, high-resolution collimator and an integrated 16-slice CT scanner was used in this study. All patients were injected with 740 MBq of 99mTc-MDP. The scan was performed in two phases, the blood pool phase (1–5 min after injection, 60 s per frame, with a total of 5 frames) and the delayed phase (3 h post-injection, single frame), with time correction for isotope decay.

Regions of interest (ROIs) were set to quantify tracer uptake on the affected and unaffected sides. Data analysis was conducted using DICOM viewing software (Version 2.10.7.103, EV Insite R, PSP Corporation, Tokyo, Japan). In this study, ROIs were manually set on the affected and unaffected sides in both the blood pool and the delayed images, and the ratio of radiotracer activity between the affected and unaffected hip joint regions was calculated.

### 2.3. Statistical Analysis

Statistical analysis was performed using GraphPad Prism 10.2.3 (GraphPad Software, San Diego, CA, USA). The normality of continuous data was assessed. Data following a normal distribution are expressed as mean ± standard deviation (x ± s), and comparisons between groups were performed using an independent samples t-test. Data without normal distribution are expressed as median (Interquartile range) [M(Q1, Q3)], and the Mann–Whitney U test was used for group comparisons. ROC curve analysis was used to evaluate the diagnostic performances of external iliac lymph node counts, bone scintigraphy thresholds, and their combined application for diagnosing PJI, calculating the area under the curve (AUC), sensitivity, and specificity. The optimal CT lymph node count threshold for distinguishing PJI from aseptic loosening was determined using the Youden J statistic (J = sensitivity + specificity − 1). The DeLong test was used to compare different diagnostic measures’ AUCs, with *p*-values less than 0.05 considered statistically significant (*p* < 0.05: *; *p* < 0.01: **; *p* < 0.001: ***). Prior to this study, sample size calculations were conducted using G*Power 3.1 software. The effect size (d) was set to 0.8, the significance level (α) to 0.05, and statistical power (1 − β) to 0.80, with a sample size allocation ratio of 2:1 between groups. The results show that to detect a moderate effect size between groups, 19 samples were required for group 1 and 39 were required for group 2, with a total of 58 samples, providing 80% statistical power at a 5% significance level.

## 3. Results

### 3.1. Baseline Characteristics of Patients

Table 1 shows the two groups’ demographic characteristics. This study included 110 patients, with 38 diagnosed with PJI according to MSIS criteria and 72 patients with aseptic failure revision. There were no significant differences between the groups in terms of age, weight, height, BMI, or gender (*p* value > 0.05).

### 3.2. Laboratory Test Results

Table 2 compares the serum CRP and ESR levels and the percentage of polymorphonuclear neutrophils (PMN%) in synovial fluid between the PJI group and the aseptic failure group. Significant differences were observed in the levels of CRP, ESR, and PMN% between the two groups (*p* value < 0.05). In the PJI group, the median ESR was 72 mm/h (range: 43–91 mm/h), significantly higher than the median ESR of 24 mm/h (range: 13–43 mm/h) in the aseptic group. The median PMN% in the synovial fluid of the PJI group was 65.65 (range: 59.67–70.15), significantly higher than the median PMN% of 61.65 (range: 54.52–69.10) in the aseptic group.

### 3.3. Counting Enlarged External Iliac Lymph Nodes on DECT

**Figure 1:**
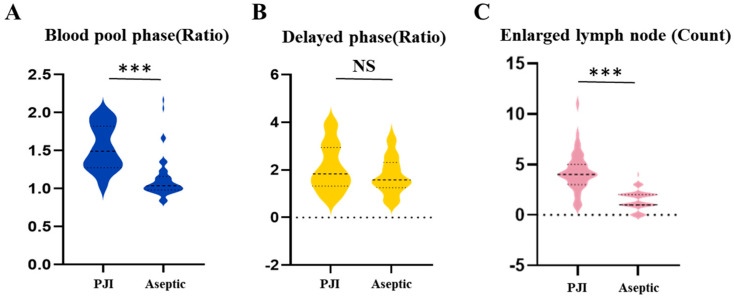
Imaging examination data. (**A**): Blood pool-phase radiotracer activity ratio between the affected and unaffected hip joints. (**B**): Delayed-phase radiotracer activity ratio between the affected and unaffected hip joints. (**C**): Count of enlarged external iliac lymph nodes. *p* < 0.001: ***; NS: no significant difference, statistically significant (*p* ≤ 0.05).

**Figure 2:**
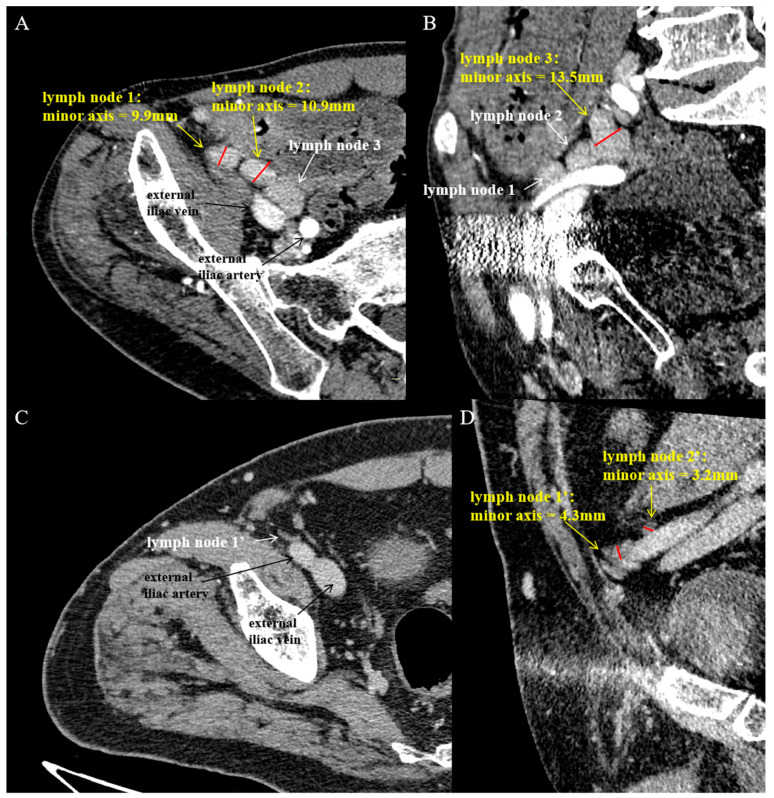
DECT examination of patients with PJI and aseptic loosening. (**A**,**B**) Male, 67 years old, diagnosed with right hip PJI; (**C**,**D**) Male, 72 years old, with right hip aseptic loosening. (**A**): An axial contrast-enhanced CT image showing the largest cross-sections of lymph node 1 and lymph node 2, with the short-axis diameter of the lymph nodes measured (red short line). (**B**): An oblique coronal contrast-enhanced CT image showing the largest cross-section of lymph node 3, with the short-axis diameter measured (red short line). (**C**): An axial contrast-enhanced CT image. (**D**): An oblique coronal contrast-enhanced CT image showing the largest cross-sections of lymph node 1′ and lymph node 2′, with the short-axis diameter measured (red short line).

DECT scanning captured images of the external iliac lymph nodes on the affected side for all patients, and the short-axis diameters of these nodes were measured (Figure 1 and Figure 2). Table 3 shows the number of enlarged external iliac lymph nodes (short-axis ≥ 5 mm) in the PJI and aseptic failure groups. The median number of enlarged lymph nodes in the PJI group was 4.0 (range: 3.0–5.0), significantly higher than the aseptic group’s median of 1.0 (range: 1.0–2.0).

### 3.4. Bone Scintigraphy

**Figure 3:**
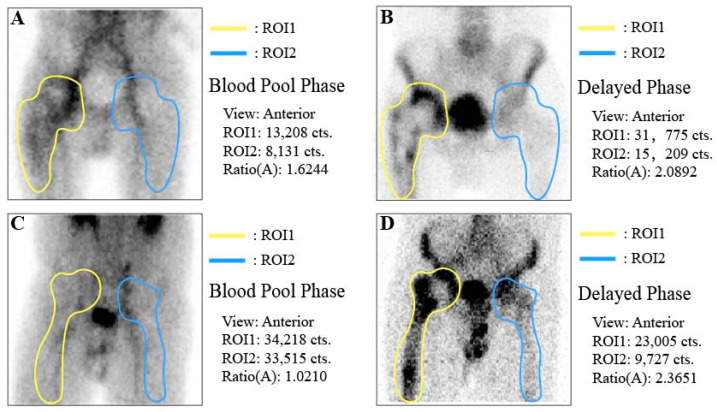
Bone scintigraphy of patients with PJI and aseptic loosening. (**A**,**B**) Male, 67 years old, diagnosed with right hip PJI; (**C**,**D**) Male, 72 years old, with right hip aseptic loosening. ROI: region of interest.

Bone scintigraphy was performed on all patients to obtain radionuclide images of both hip joints, and the ratio of radiotracer activity between the affected and unaffected sides around the hip joint was calculated (Figure 1 and Figure 3). As shown in Table 3, the blood pool-phase ratio of radiotracer activity between the affected and unaffected sides in the PJI group was significantly higher than in the aseptic group (*p* value < 0.0001), while no significant differences were observed between the two groups for the delayed phase or the ratio of the blood pool phase to the delayed phase.

**Figure 4:**
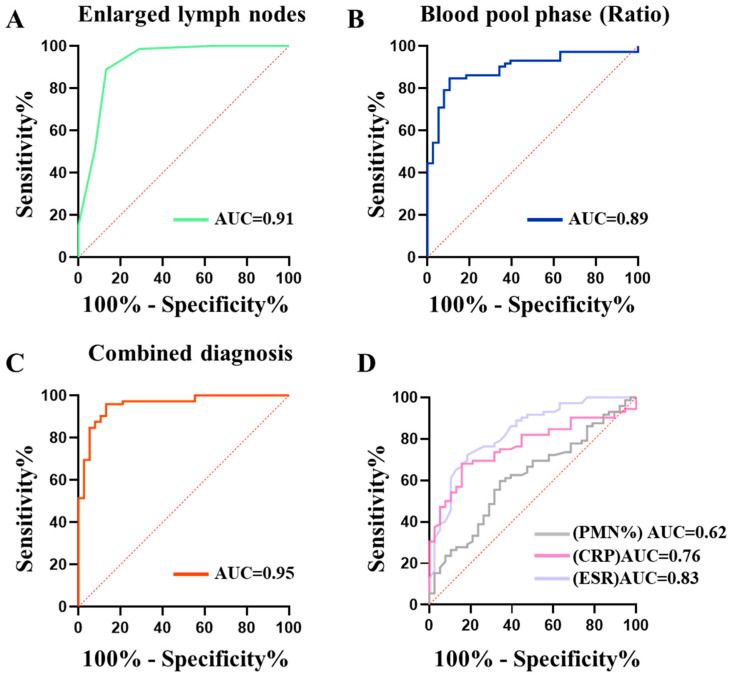
The receiver operating characteristic (ROC) curves and area under curve (AUC) for the four tests used to diagnose PJI. (**A**): ROC curve and AUC for the count of enlarged external iliac lymph nodes. (**B**): ROC curve and AUC for bone scintigraphy blood pool imaging. (**C**): ROC curve and AUC for the combination of the count of enlarged external iliac lymph nodes and bone scintigraphy blood pool imaging. (**D**): ROC curve and AUC for serological indicators CRP, ESR, and synovial fluid indicator PMN%. PMN%, polymorphonuclear cell percentage; CRP, C-reactive protein; ESR, erythrocyte sedimentation rate.

To assess the diagnostic value of the number of enlarged external iliac lymph nodes (short-axis > 5 mm) and the blood pool-phase affected/unaffected hip joint radiotracer activity ratio to distinguish between PJI and aseptic failure, we performed ROC curve analysis using the PJI group as the positive control group and the aseptic failure group as the negative control group (Figure 4). The optimal diagnostic thresholds for both the number of enlarged lymph nodes and the blood pool phase ratio were calculated (Table 4). The results showed that the number of enlarged external iliac lymph nodes and the blood pool-phase affected/unaffected hip joint radiotracer activity ratio were effective in differentiating PJI from aseptic loosening. The AUC for the number of enlarged external iliac lymph nodes was 0.91 (95% CI: 0.85–0.98), with an optimal diagnostic threshold of 2.5 nodes, a sensitivity of 88.89%, a specificity of 86.84%, and an accuracy of 88.18%. The AUC for the blood pool-phase affected/unaffected hip joint radiotracer activity ratio was 0.89 (95% CI: 0.83–0.96), with an optimal diagnostic threshold of 1.236, a sensitivity of 84.72%, a specificity of 89.47%, and an accuracy of 86.36%. The AUC for CRP was 0.76 (95% CI: 0.68–0.85), with an optimal diagnostic threshold of 7.995 mg/L, a sensitivity of 68.06%, a specificity of 81.58%, and an accuracy of 72.73%. The AUC for ESR was 0.83 (95% CI: 0.75–0.91), with an optimal diagnostic threshold of 42.50 mm/h, a sensitivity of 72.22%, a specificity of 81.58%, and an accuracy of 74.55%. The AUC for synovial fluid PMN% was 0.62 (95% CI: 0.51–0.72), with an optimal diagnostic threshold of 63.05, a sensitivity of 59.72%, a specificity of 65.79%, and an accuracy of 61.82%. Statistical analysis revealed that the AUCs for the number of enlarged external iliac lymph nodes and the blood pool-phase affected/unaffected hip joint radiotracer activity ratio were significantly higher than those of CRP, ESR, and synovial fluid PMN% (*p* value < 0.05), indicating the superior diagnostic performance of counting the number of enlarged iliac lymph nodes and the blood pool phase ratio for diagnosing PJI.

To further improve the accuracy of PJI diagnosis, we combined the number of enlarged iliac lymph nodes with the blood pool-phase affected/unaffected hip joint radiotracer activity ratio. The ROC curve analysis showed that the combined method had an AUC of 0.95 (95% CI: 0.91 to 0.99), a sensitivity of 95.83%, a specificity of 86.84%, and an accuracy of 92.73%.

## 4. Discussion

Timely and accurate diagnosis is essential for the successful treatment of PJI [25]. Orthopedic research on PJI has evolved from monitoring single indicators to combining multiple metrics, as well as from using traditional biomarkers to using advanced molecular techniques [26,27]. Yet, many of these research findings have yielded unsatisfactory results. In this prospective study, we evaluated a new imaging-based diagnostic approach for PJI treatment after hip replacement. This approach combined the counting of enlarged external iliac lymph nodes (short-axis ≥ 5 mm) with quantitative bone scintigraphy. Our study was the first to discover that the number of enlarged external iliac lymph nodes can be a sensitive marker for PJI after hip replacement. Furthermore, when combined with the blood pool-phase affected/unaffected hip joint radiotracer activity ratio in bone scintigraphy, diagnostic accuracy was further improved. Compared to traditional systemic inflammatory markers like ESR, CRP, and synovial fluid PMN%, our approach demonstrated greater sensitivity and specificity. This finding has important implications for clinical decision-making.

The lymphatic system and blood circulation exhibit crucial anatomical and functional interactions through lymph nodes [12]. External iliac lymph nodes receive lymphatic drainage from the superficial and deep inguinal lymph nodes, identifying and filtering pathogens and other foreign substances [15]. Studies suggest that during immune responses, lymph nodes may swell to 15 times their normal size [15]. However, the accuracy of a diagnosis based on a single lymph node may be affected by individual differences and other inflammatory conditions. In contrast, multiple lymph node enlargements provide a more accurate indication of local infection, a view corroborated by studies in other diseases and animal models. Tamburini et al. noted that infected mice often exhibit increased activity and multiple enlarged lymph nodes [28]. Bastuji-Garin et al. found that lymph node enlargement in multiple regions is strongly associated with cellulitis [29]. Balch et al. discovered a negative correlation between the number of enlarged lymph nodes and disease staging and prognosis in melanoma patients [30].

Despite the acknowledged strategic importance of external iliac lymph node enlargement for monitoring lower limb infections, advanced imaging techniques are required to accurately evaluate these changes. Traditional imaging techniques such as X-rays and MRI struggle to provide clear and accurate lymph node images due to interference from metal prostheses [31,32]. Though ultrasound effectively avoids interference from metal prostheses, the deep anatomical location of external iliac lymph nodes and the reflection of sound waves from prosthesis may make it difficult to accurately assess changes in this lymph node group. In contrast, DECT technology, as used in this study, employs rays of different energy levels, and algorithms recognize the attenuation characteristics of metal implants and normal tissues [33]. It processes the projection and image domains in sequence, accurately identifying and eliminating metal-induced artifacts to improve diagnostic accuracy [34,35]. A recent study by Qiang et al. similarly confirmed DECT’s diagnostic value in PJI following hip replacement surgery [36]. Moreover, DECT has few contraindications, and it is non-invasive and cost-effective, making it easier to apply in clinical practice.

Given the limitations of using lymph node enlargement as the sole diagnostic method for PJI, patients with asymptomatic extra-articular inflammatory conditions may also experience immune system activation, causing non-specific lymph node enlargement [37]. Furthermore, lymph node enlargement is closely related to changes in fluid exchange within local tissues. During infection, the synergistic effects of local metabolites and inflammatory mediators promote the leakage of plasma fluid into the interstitial space. Studies have shown that during infection, blood and lymph flow through the lymph nodes may increase by up to 25-fold, resulting in activated proliferating cell accumulation and, ultimately, lymph node enlargement [8,19]. Therefore, monitoring these dynamic fluid changes is crucial for accurately identifying infections.

Radionuclide imaging is a sensitive technique for identifying local blood flow levels in bones and joints [38]. During the 2015 European Association of Nuclear Medicine (EANM) consensus meeting, it was recommended that further nuclear medicine imaging be used to confirm PJI in suspected cases [39]. Two commonly used nuclear medicine techniques in PJI diagnosis are as follows: (1) bone scintigraphy using 99mTc-MDP; (2) radiolabeled white blood cell (WBC) scintigraphy. Though some studies indicate that WBC scintigraphy provides greater specificity and sensitivity [40], in practice, it has higher technical demands and is restricted to use in patients without a history of corticosteroid or antibiotic use. Additionally, the higher cost of WBC scintigraphy further limits its broad clinical application. As a result, we opted for bone scintigraphy using 99mTc-MDP in this study. We differentiated infection from aseptic loosening by quantifying the radiotracer activity ratio between the affected and unaffected hip joints. Similarly, using quantitative radiotracer values to improve diagnostic reproducibility and consistency has previously been confirmed in other studies [41]. Likewise, Andor W. J. M. Glaudemans et al. confirmed that quantitative indicators reduce reliance on the operator’s expertise while maintaining the test’s sensitivity and specificity [42]. In this study, we focused on the blood pool phase’s performance in bone scintigraphy and compared its diagnostic efficiency with that of the delayed phase. We discovered that the radiotracer activity ratio in the blood pool phase is more effective at distinguishing PJI from aseptic failure following hip replacement than the delayed phase. As mentioned earlier, the infection process often results in changes to local blood flow and vascular permeability. After injecting the radiotracer, we effectively captured these changes in the blood pool phase. Consistent with earlier studies, the delayed phase’s quantitative metrics did not distinguish between infection and aseptic loosening, likely due to widespread bone resorption.

## 5. Conclusions

This study demonstrates that combining the counting of enlarged external iliac lymph nodes with the quantification of the hip joint uptake ratio in bone scintigraphy represents a sensitive and accurate method for the early diagnosis of periprosthetic joint infection (PJI) after hip replacement. Compared to traditional systemic inflammatory markers such as ESR, CRP, and synovial PMN%, our approach exhibits higher sensitivity and specificity.

## Figures and Tables

**Table 1:** The demographic data of the study population.

Characteristic	PJI Group (*N* = 38)	Aseptic Failure (*N* = 72)	*p*-Value
Age (years)	65.29 ± 12.79	66.29 ± 12.68	0.695
Weight (kg)	60.77 ± 10.99	59.45 ± 11.20	0.554
Height (cm)	161.31 ± 8.69	160.65 ± 9.45	0.720
BMI (kg/m^2^)	23.23 ± 2.99	22.92 ± 3.55	0.640
**Gender**			0.756
Male	22 (57.89%)	38 (52.78%)	
Female	16 (42.11%)	34 (47.22%)	

Variables are expressed as mean ± SD or numbers (percentage). BMI, body mass index; PJI, periprosthetic joint infections. Statistically significant (*p* ≤ 0.05).

**Table 2:** Inflammatory markers in patients with PJI and aseptic revision.

Inflammatory Marker	PJI Group (*N* = 38)	Aseptic Failure (*N* = 72)	*p*-Value
CRP (mg/L)	1.49 (1.29, 1.81)	1.04 (0.98, 1.15)	<0.001
ESR (mm/h)	1.84 (1.38, 2.89)	1.58 (1.26, 2.28)	<0.001
PMN%	65.65 (59.67, 70.15)	61.65 (54.52, 69.10)	<0.05

CRP, C-reactive protein; ESR, erythrocyte sedimentation rate; PMN%, polymorphonuclear cell percentage. Statistically significant (*p* ≤ 0.05).

**Table 3:** Imaging Examination in patients with PJI and aseptic revision.

Imaging Examination	PJI Group (*N* = 38)	Aseptic Failure (*N* = 72)	*p*-Value
Blood phase (Ratio)	1.49 (1.29, 1.81)	1.04 (0.98, 1.15)	<0.001
Delay phase (Ratio)	1.84 (1.38, 2.89)	1.58 (1.26, 2.28)	0.142
Lymph node (N)	4.0 (3.0, 5.0)	1.5(1.0, 2.0)	<0.001

PJI, periprosthetic joint infections; statistically significant (*p* ≤ 0.05).

**Table 4:** Performance parameters of diagnostic PJI methods.

Diagnosis Method	AUC	Sensitivity, % (95% CI)	Specificity, % (95% CI)	Cutoff Value	PPV, %	NPV, %	Accuracy, %
Blood pool phase (ratio)	0.89 (0.83 to 0.96)	84.72 (74.68 to 91.25)	89.47 (75.87 to 95.83)	1.236	75.56	93.85	86.36
Enlarged lymph nodes	0.91 (0.85 to 0.98)	88.89 (79.58 to 94.26)	86.84 (72.67 to 94.25)	2.500	80.49	94.12	88.18
Combined diagnosis	0.95 (0.91 to 0.99)	95.83 (88.45 to 98.86)	86.84(72.67 to 94.25)	\	91.67	93.24	92.73
ESR	0.83 (0.75 to 0.91)	72.22 (60.95 to 81.58)	81.58 (66.58 to 90.78)	42.50	60.00	86.67	74.55
CRP	0.76 (0.68 to 0.85)	68.06 (56.61 to 77.67)	81.58 (66.58 to 90.78)	7.995	57.41	87.50	72.73
PMN%	0.62 (0.51 to 0.72)	59.72 (48.18 to 70.28)	65.79 (49.89 to 78.79)	63.05	46.30	75.44	61.82

CRP, C-reactive protein; ESR, erythrocyte sedimentation rate; PMN%, polymorphonuclear cell percentage; CI, confidence interval; PJI, periprosthetic joint infection; PPV, positive predictive value; NPV, negative predictive value.

## Data Availability

For privacy and security reasons, the data presented in this study are available on request from the corresponding author.

## References

[1] Qin L., Yang S., Zhao C., Yang J., Li F., Xu Z., Yang Y., Zhou H., Li K., Xiong C. (2024). Prospects and challenges for the application of tissue engineering technologies in the treatment of bone infections. Bone Res..

[2] Zhou H., Yang Y., Zhang Y., Li F., Shen Y., Qin L., Huang W. (2024). Current Status and Perspectives of Diagnosis and Treatment of Periprosthetic Joint Infection. Infect. Drug Resist..

[3] Bingham J.S., Hassebrock J.D., Christensen A.L., Beauchamp C.P., Clarke H.D., Spangehl M.J. (2020). Screening for Periprosthetic Joint Infections with ESR and CRP: The Ideal Cutoffs. J. Arthroplast..

[4] Huerfano E., Bautista M., Huerfano M., Bonilla G., Llinas A. (2017). Screening for Infection Before Revision Hip Arthroplasty: A Meta-analysis of Likelihood Ratios of Erythrocyte Sedimentation Rate and Serum C-reactive Protein Levels. J. Am. Acad. Orthop. Surg..

[5] Wouthuyzen-Bakker M. (2023). Cultures in periprosthetic joint infections, the imperfect gold standard?. EFORT Open Rev..

[6] Hong H.-L., Flurin L., Thoendel M.J., Wolf M.J., Abdel M.P., E Greenwood-Quaintance K., Patel R. (2023). Targeted Versus Shotgun Metagenomic Sequencing-based Detection of Microorganisms in Sonicate Fluid for Periprosthetic Joint Infection Diagnosis. Clin. Infect. Dis..

[7] Mei J., Hu H., Zhu S., Ding H., Huang Z., Li W., Yang B., Zhang W., Fang X. (2023). Diagnostic Role of mNGS in Polymicrobial Periprosthetic Joint Infection. J. Clin. Med..

[8] Melo-Silva C.R., Sigal L.J. (2024). Innate and adaptive immune responses that control lymph-borne viruses in the draining lymph node. Cell. Mol. Immunol..

[9] Grasso C., Pierie C., Mebius R., van Baarsen L. (2021). Lymph node stromal cells: Subsets and functions in health and disease. Trends Immunol..

[10] Wong E., Montoya B., Stotesbury C., Ferez M., Xu R.-H., Sigal L.J. (2019). Langerhans Cells Orchestrate the Protective Antiviral Innate Immune Response in the Lymph Node. Cell Rep..

[11] Wong E., Xu R.-H., Rubio D., Lev A., Stotesbury C., Fang M., Sigal L.J. (2018). Migratory Dendritic Cells, Group 1 Innate Lymphoid Cells, and Inflammatory Monocytes Collaborate to Recruit NK Cells to the Virus-Infected Lymph Node. Cell Rep..

[12] Piersma S.J. (2024). Tissue-specific features of innate lymphoid cells in antiviral defense. Cell. Mol. Immunol..

[13] Ghirardelli M.L., Jemos V., Gobbi P.G. (1999). Diagnostic approach to lymph node enlargement. Haematologica.

[14] Qin L., Zhao C., Wang H., Yang J., Chen L., Su X., Wei L., Zhang T., Li J., Jian C. (2023). Detection of inguinal lymph nodes is promising for the diagnosis of periprosthetic joint infection. Front. Cell. Infect. Microbiol..

[15] Xiao Y.-T., Zhao X., Chang Y., Lu X., Wang Y., Zhang H., Ren S. (2020). Assessing the safety and feasibility of neoadjuvant hormone and radiation therapy followed by robot-assisted radical prostatectomy for treating locally advanced prostate cancer: Protocol for an open-label, dose-escalation, single-centre, phase I clinical trial. BMJ Open.

[16] Qu Y., Frazer L.C., O’Connell C.M., Tarantal A.F., Andrews C.W., O’Connor S.L., Russell A.N., Sullivan J.E., Poston T.B., Vallejo A.N. (2015). Comparable Genital Tract Infection, Pathology, and Immunity in Rhesus Macaques Inoculated with Wild-Type or Plasmid-Deficient Chlamydia trachomatis Serovar D. Infect. Immun..

[17] Isern-Kebschull J., Tomas X., García-Díez A.I., Morata L., Ríos J., Soriano A. (2019). Accuracy of Computed Tomography–Guided Joint Aspiration and Computed Tomography Findings for Prediction of Infected Hip Prosthesis. J. Arthroplast..

[18] Bontumasi N., Jacobson J.A., Caoili E., Brandon C., Kim S.M., Jamadar D. (2014). Inguinal lymph nodes: Size, number, and other characteristics in asymptomatic patients by CT. Surg. Radiol. Anat..

[19] de Casas P.C., Knöpper K., Sarkar R.D., Kastenmüller W. (2023). Same yet different—How lymph node heterogeneity affects immune responses. Nat. Rev. Immunol..

[20] Ouyang Z., Li H., Liu X., Zhai Z., Li X. (2014). Prosthesis infection: Diagnosis after total joint arthroplasty with three-phase bone scintigraphy. Ann. Nucl. Med..

[21] Romanò C.L., Petrosillo N., Argento G., Sconfienza L.M., Treglia G., Alavi A., Glaudemans A.W.J.M., Gheysens O., Maes A., Lauri C. (2020). The Role of Imaging Techniques to Define a Peri-Prosthetic Hip and Knee Joint Infection: Multidisciplinary Consensus Statements. J. Clin. Med..

[22] Araki Y., Yamamoto N., Hayashi K., Takeuchi A., Miwa S., Igarashi K., Higuchi T., Abe K., Taniguchi Y., Yonezawa H. (2023). A Viability Analysis of Tumor-Bearing Frozen Autograft for the Reconstruction After Resection of Malignant Bone Tumors Using 99mTc-MDP Scintigraphy. Clin. Nucl. Med..

[23] Parvizi J., Gehrke T., International Consensus Group on Periprosthetic Joint Infection (2014). Definition of Periprosthetic Joint Infection. J. Arthroplast..

[24] Wyngaert T.V.D., Strobel K., Kampen W.U., Kuwert T., van der Bruggen W., Mohan H.K., Gnanasegaran G., Delgado-Bolton R., Weber W.A., On behalf of the EANM Bone & Joint Committee and the Oncology Committee (2016). The EANM practice guidelines for bone scintigraphy. Eur. J. Nucl. Med..

[25] Saavedra-Lozano J., Falup-Pecurariu O., Faust S.N., Girschick H., Hartwig N., Kaplan S., Lorrot M., Mantadakis E., Peltola H., Rojo P. (2017). Bone and Joint Infections. Pediatr. Infect. Dis. J..

[26] Qin L., Hu N., Zhang Y., Yang J., Zhao L., Zhang X., Yang Y., Zhang J., Zou Y., Wei K. (2023). Antibody-antibiotic conjugate targeted therapy for orthopedic implant-associated intracellular S. aureus infections. J. Adv. Res..

[27] Qin L., Li F., Gong X., Wang J., Huang W., Hu N. (2020). Combined Measurement of D-Dimer and C-Reactive Protein Levels: Highly Accurate for Diagnosing Chronic Periprosthetic Joint Infection. J. Arthroplast..

[28] Tamburini B.A., Burchill M.A., Kedl R.M. (2014). Antigen capture and archiving by lymphatic endothelial cells following vaccination or viral infection. Nat. Commun..

[29] Dupuy A., Benchikhi H., Roujeau J.-C., Bernard P., Vaillant L., Chosidow O., Sassolas B., Guillaume J.-C., Grob J.-J., Bastuji-Garin S. (1999). Risk factors for erysipelas of the leg (cellulitis): Case-control study. BMJ.

[30] Lange J.R., Palis B.E., Chang D.C., Soong S.-J., Balch C.M. (2007). Melanoma in Children and Teenagers: An Analysis of Patients from the National Cancer Data Base. J. Clin. Oncol..

[31] Picchio M., Mapelli P., Panebianco V., Castellucci P., Incerti E., Briganti A., Gandaglia G., Kirienko M., Barchetti F., Nanni C. (2015). Imaging biomarkers in prostate cancer: Role of PET/CT and MRI. Eur. J. Nucl. Med. Mol. Imaging.

[32] Di Muzio N., Fodor A., Berardi G., Mapelli P., Gianolli L., Messa C., Picchio M. (2012). Lymph nodal metastases: Diagnosis and treatment. Q. J. Nucl. Med. Mol. Imaging.

[33] Jardon M., Fritz J., Samim M. (2024). Imaging approach to prosthetic joint infection. Skelet. Radiol..

[34] Moore J., Remy J., Altschul E., Chusid J., Flohr T., Raoof S., Remy-Jardin M. (2024). Thoracic Applications of Spectral CT Scan. Chest.

[35] Boudabbous S., Paulin E.N., Delattre B.M.A., Hamard M., Vargas M.I. (2021). Spinal disorders mimicking infection. Insights Imaging.

[36] Cheng Q., Yang Y., Li F., Li X., Qin L., Huang W. (2024). Dual-Energy Computed Tomography Iodine Maps: Application in the Diagnosis of Periprosthetic Joint Infection in Total Hip Arthroplasty. J. Arthroplast..

[37] Senger J.-L.B., Kadle R.L., Skoracki R.J. (2023). Current Concepts in the Management of Primary Lymphedema. Medicina.

[38] Gemmel F., van den Wyngaert H., Love C., Welling M.M., Gemmel P., Palestro C.J. (2012). Prosthetic joint infections: Radionuclide state-of-the-art imaging. Eur. J. Nucl. Med. Mol. Imaging.

[39] Jutte P., Lazzeri E., Sconfienza L.M., Cassar-Pullicino V., Trampuz A., Petrosillo N., Signore A. (2014). Diagnostic flowcharts in osteomyelitis, spondylodiscitis and prosthetic joint infection. Q. J. Nucl. Med. Mol. Imaging.

[40] Palestro C.J. (2023). Molecular Imaging of Periprosthetic Joint Infections. Semin. Nucl. Med..

[41] Gandsman E., McCullough R. (1990). Dynamic bone imaging in the differential diagnosis of skeletal lesions. Int. J. Radiat. Appl. Instrum. Part B Nucl. Med. Biol..

[42] Glaudemans A.W., De Vries E.F., Vermeulen L.E., Slart R.H., Dierckx R.A., Signore A. (2013). A large retrospective single-centre study to define the best image acquisition protocols and interpretation criteria for white blood cell scintigraphy with 99mTc-HMPAO-labelled leucocytes in musculoskeletal infections. Eur. J. Nucl. Med. Mol. Imaging.

